# Molecular and Cellular Basis of Microvascular Perfusion Deficits Induced by *Clostridium perfringens* and *Clostridium septicum*

**DOI:** 10.1371/journal.ppat.1000045

**Published:** 2008-04-11

**Authors:** Michael J. Hickey, Rain Y. Q. Kwan, Milena M. Awad, Catherine L. Kennedy, Lauren F. Young, Pam Hall, Leanne M. Cordner, Dena Lyras, John J. Emmins, Julian I. Rood

**Affiliations:** 1 Centre for Inflammatory Diseases, Monash University Department of Medicine, Monash Medical Centre, Clayton, Victoria, Australia; 2 Australian Bacterial Pathogenesis Program, Department of Microbiology, Monash University, Victoria, Australia; 3 Department of Immunology, Monash University, Alfred Medical Research and Education Precinct, Prahran, Victoria, Australia; Schepens Eye Research Institute, United States of America

## Abstract

Reduced tissue perfusion leading to tissue ischemia is a central component of the pathogenesis of myonecrosis caused by *Clostridium perfringens*. The *C. perfringens* α-toxin has been shown capable of inducing these changes, but its potential synergy with perfringolysin O (θ-toxin) is less well understood. Similarly, *Clostridium septicum* is a highly virulent causative agent of spontaneous gas gangrene, but its effect on the microcirculation has not been examined. Therefore, the aim of this study was to use intravital microscopy to examine the effects of *C. perfringens* and *C. septicum* on the functional microcirculation, coupled with the use of isogenic toxin mutants to elucidate the role of particular toxins in the resultant microvascular perfusion deficits. This study represents the first time this integrated approach has been used in the analysis of the pathological response to clostridial toxins. Culture supernatants from wild-type *C. perfringens* induced extensive cell death within 30 min, as assessed by *in vivo* uptake of propidium iodide. Furthermore, significant reductions in capillary perfusion were observed within 60 min. Depletion of either platelets or neutrophils reduced the alteration in perfusion, consistent with a role for these blood-borne cells in obstructing perfusion. In addition, mutation of either the α-toxin or perfringolysin O structural genes attenuated the reduction in perfusion, a process that was reversed by genetic complementation. *C. septicum* also induced a marked reduction in perfusion, with the degree of microvascular compromise correlating with the level of the *C. septicum* α-toxin. Together, these data indicate that as a result of its ability to produce α-toxin and perfringolysin O, *C. perfringens* rapidly induces irreversible cellular injury and a marked reduction in microvascular perfusion. Since *C. septicum* induces a similar reduction in microvascular perfusion, it is postulated that this function is central to the pathogenesis of clostridial myonecrosis, irrespective of the causative bacterium.

## Author Summary

Clostridial myonecrosis is a life-threatening process induced by infection with species such as *C. perfringens* and *C. septicum*. The associated pathology includes muscle death and a characteristic disruption in tissue perfusion. Exotoxins produced by these species have been implicated in the reduction in perfusion. However, how these toxins function in tandem remains unclear. In this study we used intravital microscopy to study microvascular blood flow in a muscle exposed to products of *C. perfringens* and *C. septicum*. *C. perfringens* supernatants induced cellular injury and a progressive reduction in blood flow. Removal of blood-borne platelets and neutrophils from the circulation reduced the alteration in blood flow. In addition, this response was reduced by genetic deletion of either the α-toxin or perfringolysin O, providing the first indication that each of these exotoxins contributes to the reduction in blood supply to affected tissues. Using a similar approach, we observed that *C. septicum* supernatant induced a comparable reduction in perfusion, which was mediated in part via the *C. septicum* α-toxin. These results indicate that platelets, neutrophils and multiple clostridial toxins contribute to reduced blood supply and oxygen delivery associated with clostridial infection and suggest that the dominant component of the pathology is toxin-induced cellular injury and death.

## Introduction

Gas gangrene is a life-threatening syndrome most commonly associated with invasion of tissue by the anaerobic bacterium, *Clostridium perfringens* type A. The pathology of gas gangrene is highly complex, but is thought to be mediated by disruptions in tissue perfusion, associated with alterations in platelet aggregation and leukocyte margination [1],[2]. Histological assessment of infected tissues, both in humans and experimental animals, has revealed a characteristic pattern of extensive myonecrosis, edema, thrombosis, and restriction of leukocyte infiltration to the perivascular regions in the infected site [1],[3]. Toxins produced by *C. perfringens* type A have been shown to be essential to the development of this pathology [2]-[7]. α-toxin and perfringolysin O are the major toxins produced by type A strains but they are known to produce other extracellular toxins and enzymes including a collagenase, the cysteine protease α-clostripain, sialidases and hyaluronidases [8],[9]. Toxin production in *C. perfringens* is regulated in response to environmental or growth phase signals by the VirSR two-component signal transduction system [9].

Preparations containing the *C. perfringens* α-toxin, which has phospholipase C and sphingomyelinase activity [10], and perfringolysin O, a cholesterol-dependent cytolysin, also known as θ-toxin [11], have been shown to induce rapid and irreversible reductions in muscle blood flow, via generation of intravascular platelet-leukocyte aggregates [6]. *In vivo* and *in vitro* studies implicate platelet adhesion glycoproteins GPIIb/IIIa and P-selectin (CD62P) as contributing to these heterotypic aggregates. However, the precise mechanism of action of these toxins, particularly in combination, requires further investigation.

Examination of the vascular effects of purified clostridial toxins has generated findings that emphasize the complexity of the tissue response. Purified α-toxin has been shown to promote myonecrosis and disrupted perfusion within minutes of application to a muscle, responses which correlate with the histopathological appearance of affected tissue 7,12. Furthermore, mutation of the α-toxin gene, *plc*, is associated with more effective leukocyte recruitment to the site of *C. perfringens* infection, suggesting a critical role for this toxin in limiting leukocyte recruitment [3],[4]. In contrast, α-toxin has been shown to promote adhesion of leukocytes to cultured endothelial cells and to induce leukocyte recruitment and expression of endothelial adhesion molecules *in vivo* [13]. Similarly, mutation of the perfringolysin O structural gene, *pfoA*, has been shown to allow increased leukocytic infiltration into infected tissues [3]. These findings suggest that the α-toxin alone is insufficient to account for the inability of leukocytes to efficiently access *C. perfringens*-infected tissue and raise the possibility that the complete complement of *C. perfringens* toxins is necessary for the most damaging response. This concept is supported by several studies that indicate that α-toxin and perfringolysin O act synergistically, such that the most severe pathology is only observed if both toxins are present [3],[5],[14]. If so, then experiments with purified toxins may not accurately reflect the complex pathology associated with clostridial infection.

Virulence studies in the mouse myonecrosis model using mutant *C. perfringens* strains lacking α-toxin and perfringolysin O have provided clear evidence of the roles of these toxins in promoting myonecrosis [3]–[5]. However, the effects of these mutant strains on myonecrosis are yet to be correlated with effects on the muscle microcirculation, as assessed using real-time *in vivo* imaging. Moreover, another clostridial species, *Clostridium septicum,* can also cause myonecrotic tissue infections, including atraumatic or spontaneous gas gangrene [15],[16]. *C. septicum* also produces an α-toxin, which does not have phospholipase C activity, and is not related to the α-toxin of *C. perfringens*; it is a pore-forming cytolysin encoded by the *csa* gene. The virulence of *C. septicum* is chiefly attributable to its α-toxin [17]. However, the pathological effects of *C. septicum* on the microcirculation, and the potential function of the α-toxin in this area, have not been examined. Therefore, the aim of this study was to investigate the cellular and molecular basis of vascular disruption associated with exposure to products of clostridial strains capable of inducing myonecrotic infections. This objective was achieved using intravital microscopy to quantitate microvascular perfusion and cellular injury during exposure to supernatants from cultures of *C. perfringens* and *C. septicum*. Use of mutant clostridial strains deficient in various toxins enabled the relative roles of these gene products to be assessed. This study represents the first time this integrated approach has been used in the analysis of the pathological response to toxins released by *C. perfringens* and *C. septicum*.

## Results

### Mechanisms of *C. perfringens*-mediated reduction in microvascular perfusion

**Figure 1:**
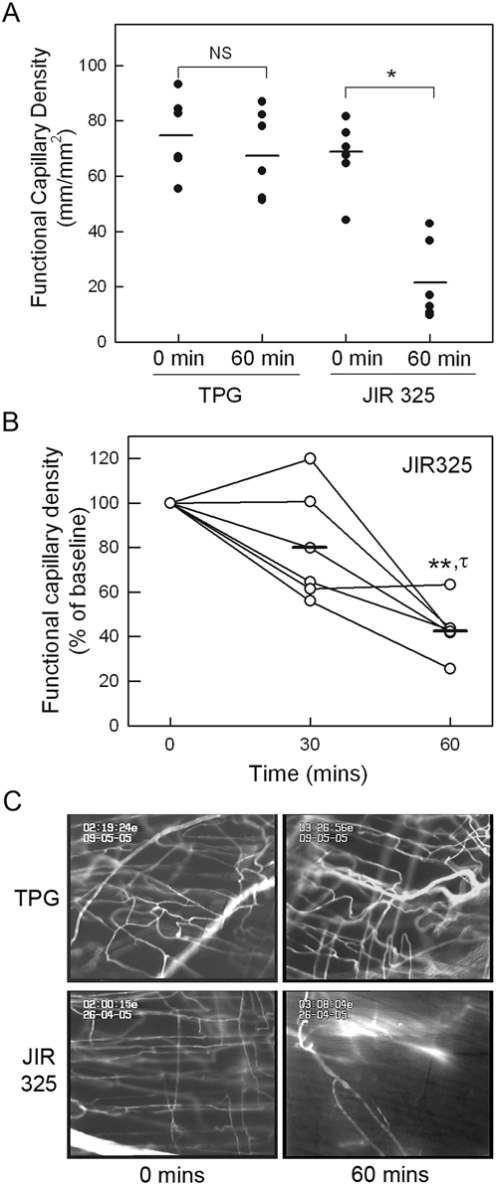
Effect of *C. perfringens* supernatant on microvascular perfusion in the mouse cremaster muscle. A: Functional capillary density was assessed in cremaster muscles under basal conditions, and after 60 min of superfusion with either TPG medium (as control, n = 6) or filtered supernatant from *C. perfringens* (JIR325, n = 6). Each data point indicates an individual animal. Horizontal bar denotes group mean. * denotes *p*<0.001 versus basal data using paired *t*-test. NS denotes not significant. B: Time course of reduction in perfusion in muscles exposed to *C. perfringens* supernatant. Microvascular perfusion was quantitated prior to, and after 30 and 60 min exposure to JIR325 supernatant. Data are displayed as the 30 & 60 min functional capillary density readings expressed as a percentage of the baseline reading for each individual animal. ** denotes *p*<0.01 versus baseline data, and τ denotes *p*<0.02 versus 30 min (using paired *t*-test). C: Representative images displaying microvascular perfusion, as demonstrated by presence of infused sodium fluorescein within microvessels. Images are shown for mice exposed to either TPG or JIR325 supernatants, both prior to and after 60 min of supernatant exposure.

In initial experiments, we quantitated the effect of culture supernatants from a wild-type, virulent *C. perfringens* type A strain, JIR325, on microvascular perfusion in the cremaster muscle. Prior to commencing supernatant superfusion, functional capillary density averaged approximately 75 mm/mm^2^ (**Figure 1**). After 60 min superfusion with the Trypticase-peptone glucose (TPG) culture medium (diluted 1:1 in bicarbonate superfusion buffer), microvascular perfusion was not significantly altered from basal levels (*p* = 0.2). In contrast, superfusion with similarly diluted culture supernatant from strain JIR325 caused a marked reduction in microvascular perfusion. After 60 min, functional capillary density was significantly reduced (*p*<0.001) to 15-40% of the level at the start of the experiment (**Figure 1A**). Additional experiments were performed to examine the time course of the reduction in capillary perfusion in more detail. After 30 min exposure to *C. perfringens* supernatant, mean functional capillary density was not significantly reduced from baseline levels (**Figure 1B**), although in some animals at this time, blood flow in cremasteric arterioles was inconsistent and pulsatile, and regions of arteriolar constriction were apparent (data not shown). In contrast, by 60 min, perfusion was significantly reduced relative to both 0 and 30 mins.

**Figure 1C** shows representative images of the microcirculation after 60 min superfusion with either TPG or JIR325 supernatant, demonstrating perfused microvessels labeled with sodium fluorescein, and the severe reduction in microvascular perfusion after 60 min exposure to supernatant from JIR325. It was notable that in supernatant-exposed tissue, fluorescein remained visible in larger arteries, but was rarely observed in capillaries, indicating that smallest elements of the vasculature were most affected by the defect in perfusion at this time point. Videos showing microvascular perfusion in representative experiments from mice exposed to TPG (**Video S1**) or JIR325 (**Video S2**) are available on-line in the *Supporting Information* section.

**Figure 2:**
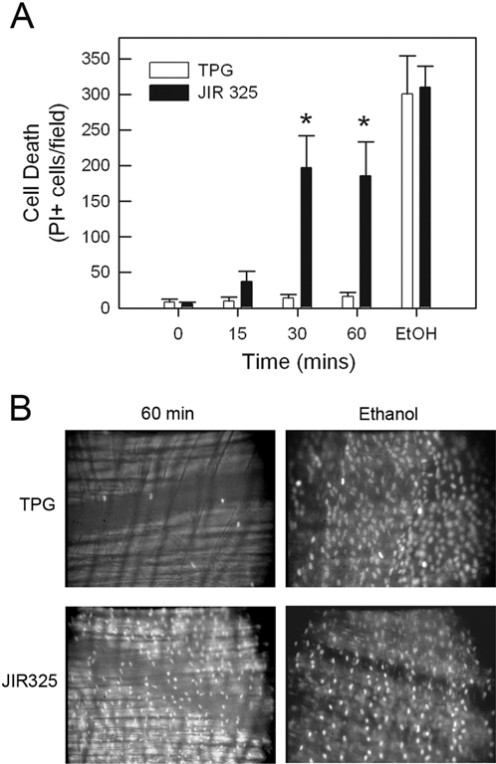
Effect of *C. perfringens* supernatant on cell viability in the mouse cremaster muscle. A: Cell death was assessed via propidium iodide (PI) staining in cremaster muscles superfused with either TPG medium (as control, n = 3) or filtered supernatant from *C. perfringens* (JIR325, n = 3). Images were captured at 0, 15, 30 and 60 min of superfusion. To assess the validity of this approach and determine the maximal degree of PI staining, at the end of the experiment muscles were treated with absolute ethanol and PI staining re-assessed. B: Representative images of PI-stained muscles superfused with either TPG or JIR325, taken (i) after 60 min supernatant superfusion, and (ii) after subsequent ethanol superfusion. * denotes *p*<0.05 versus TPG-treated animals.

To assess the possibility that the reduction in microvascular perfusion induced by *C. perfringens* supernatant was associated with cell death, propidium iodide (PI) staining was used to identify irrevocably injured cells in the cremaster muscle. Exposure of the tissue to absolute ethanol at the end of the experiment demonstrated that this staining approach was capable of identifying dead cells (**Figure 2A, B**). Superfusion with TPG medium induced minimal cell death (**Figure 2**). However, in muscles superfused with JIR325 supernatant, the number of PI-stained cells was significantly increased within 30 min, with no further increase observed at 60 min (**Figure 2A**). Notably, the number of PI-stained cells observed after treatment with JIR325 was greater than 50% of those ultimately stained following exposure of the tissue to ethanol (**Figure 2A, B**). Therefore, it was concluded that treatment with *C. perfringens* culture supernatant led to substantial cell death.

**Figure 3:**
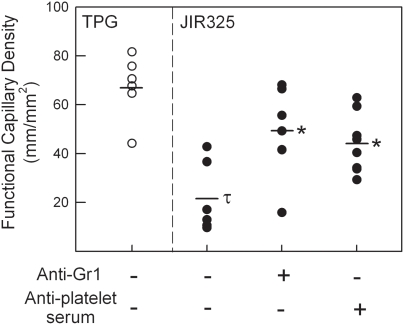
Roles of neutrophils and platelets in *C. perfringens*-induced perfusion defect. Groups of mice were either left untreated (n = 6), or depleted of either neutrophils (*anti-Gr1*, n = 5) or platelets (*anti-platelet serum*, n = 8) prior to exposure of the cremaster muscle to *C. perfringens* (JIR325) supernatant. Subsequently, functional capillary density was quantitated 60 min after commencing superfusion. Data are also shown for mice following 60 min superfusion of TPG alone (first column). τ denotes *p*<0.05 versus TPG. * denotes *p*<0.01 versus JIR325 alone.

Previous studies have implicated alterations in neutrophil margination and platelet aggregation in *C. perfringens*-mediated blood flow alterations [7],[18]. To assess these factors in this model system, mice were depleted of either neutrophils or platelets, using anti-Gr-1 or anti-platelet serum respectively, prior to undergoing exposure to culture supernatant. These approaches have been validated previously in this laboratory as achieving greater than 95% depletion of their respective targets over a similar time course to that used in the current experiment [19]. In mice depleted of either neutrophils or platelets, the reduction in microvascular perfusion induced by JIR325 supernatant was significantly attenuated (**Figure 3**). These data directly implicate these blood-borne cells in contributing to *C. perfringens*-induced microvascular collapse.

**Figure 4:**
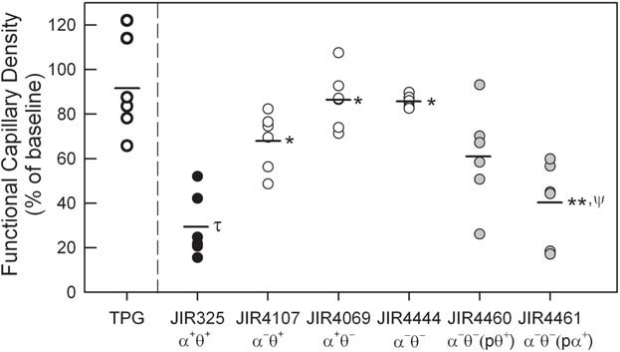
Comparison of perfusion defect induced by supernatants from genetically altered *C. perfringens* strains. Supernatants were generated from the following *C. perfringens* strains: wild-type JIR325 (α^+^θ^+^); α-toxin-deficient JIR4107 (α^−^θ^+^); perfringolysin O-deficient JIR4069 (α^+^θ^−^); double toxin-deficient JIR4444 (α^−^θ^−^); perfringolysin O-complemented JIR4460/α^−^θ^−^(pθ^+^) (where p denotes gene inserted via a shuttle plasmid); and α-toxin-complemented JIR4461/α^−^θ^−^(pα^+^) (n = 6/group). Supernatants were applied to the cremaster muscle as previously described. Functional capillary density measurements were made prior to, and after 60 min of supernatant superfusion. Data are displayed as the 60 min functional capillary density readings expressed as a percentage of the baseline reading for each individual animal. Data are also shown for TPG alone (first column). * denotes *p*<0.001 versus JIR325. τ denotes *p*<0.05 versus TPG. ** denotes *p*<0.05 versus JIR4444. ψ denotes NS between JIR325 and JIR4461.

To determine which *C. perfringens* toxin(s) were responsible for the reduction in microvascular perfusion, supernatants were prepared from isogenic *C. perfringens* strains genetically deficient in the production of either α-toxin (*plc* mutant, JIR4107) or perfringolysin O (*pfoA* mutant, JIR4069) [4] or both toxins (JIR4444) [5]. The toxin levels for each of these strains are shown in **Table 1**, confirming the absence of activity of the respective toxins. However, it is notable that the α-toxin activity in the perfringolysin O-mutant JIR4069 was lower than that in the wild-type strain (**Table 1**). Animals treated with α-toxin-deficient supernatant (JIR4107) displayed a significant attenuation in the normal *C. perfringens*-induced perfusion deficit (**Figure 4**). Similarly, supernatants from the perfringolysin O mutant (JIR4069) resulted in an almost complete abolition of the microvascular collapse seen in response to superfusion with supernatants from wild-type *C. perfringens* (**Figure 4**). The absence of both α-toxin and perfringolysin O (JIR4444) resulted in a minimal reduction in microvascular perfusion (**Figure 4**) & (**Video S3**, *Supporting Information* section), comparable to that observed following superfusion with medium alone (**Figure 1**). These data suggest that both α-toxin and perfringolysin O are capable of inducing microvascular perfusion collapse within 60 minutes.

**Table 1:** Relative toxin activities in supernatants from wild-type and mutant *C. perfringens* cultures

Strain	Genotype	Ref	Toxin status^a^	α-toxin (units mg^−1^ protein) (x 10^3^)	Perfringolysin O (log_2_ (titre))
JIR325^b^	Wild-type	[34]	Plc^+^ PFO^+^	22.7±3.9^c^	6.5±0.3
JIR4107	JIR325*plc::ermB*	[4]	Plc^−^ PFO^+^	<0.9^d^	5.7±0.1
JIR4069	JIR325*pfoA::ermB*	[4]	Plc^+^ PFO^−^	9.1±4.5	<1.0^d^
JIR4444	JIR4069 *plc*Ω*pJIR1774*	[5]	Plc^−^ PFO^−^	<0.9	<1.0
JIR4460	JIR4444(pJIR871)	[5]	Plc^−^ PFO^+^	<0.9	6.5±0.1
JIR4461	JIR4444(pJIR1642)	[5]	Plc^+^ PFO^−^	18.1±2.7	<1.0

a Plc signifies α-toxin, PFO signifies perfringolysin O

b JIR325 is a rifampicin and nalidixic acid resistant derivative of strain 13, a human gangrene isolate.

c Data are shown as mean±SEM of at least two independent cultures

d Limit of detectability

The roles of the individual *C. perfringens* toxins were investigated in more detail using supernatants from complemented *C. perfringens* strains. These strains were generated from the double-mutant JIR4444 such that either perfringolysin O (JIR4460) or α-toxin (JIR4461) were now expressed [5]. Assays of toxin activity in these strains confirmed complementation of the respective toxin to levels comparable to those in the wild-type strain (**Table 1**). In this assay, complementation with the α-toxin resulted in a significant reduction in perfusion relative to that seen with supernatants from the double mutant (JIR4444) (**Figure 4**). Indeed, the reduction in perfusion induced by JIR4461-derived, α-toxin^+^ supernatants was not statistically different from that induced by supernatants from wild-type *C. perfringens* (JIR325). In contrast, complementation with perfringolysin O alone resulted in a variable response which was not significantly altered from that seen in response to the double mutant (**Figure 4**).

### *C. septicum* supernatants also cause reduction in microvascular perfusion

**Figure 5:**
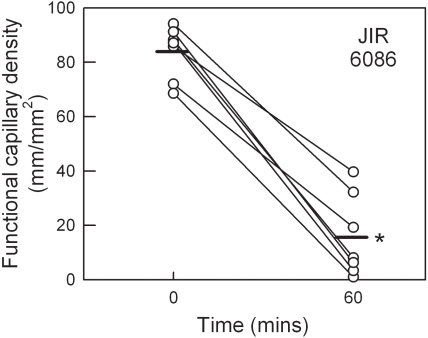
Effect of *C. septicum* supernatant on microvascular perfusion in the mouse cremaster muscle. Functional capillary density was assessed in cremaster muscles prior to, and after 60 min of superfusion with filtered supernatant from *C. septicum* (JIR6086, n = 7). * denotes *p*<0.001 versus basal data using paired *t*-test.

**Figure 6:**
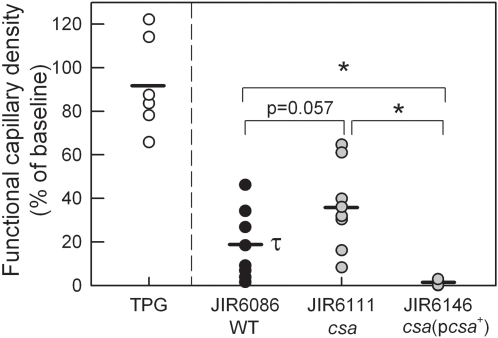
Role of the *C. septicum* α-toxin in reducing muscle microvascular perfusion. Supernatants were prepared from wild-type *C. septicum* (JIR6086/WT), or genetically-modified *C. septicum* strains either lacking the *C. septicum* α-toxin (JIR6111/*csa*, n = 7), or complemented with the α-toxin (JIR6146/*csa*(p*csa* ^+^), n = 3). Supernatants were applied to the muscle as previously described. Functional capillary density measurements were made prior to, and after 60 min of supernatant superfusion. Data are displayed as the 60 min functional capillary density readings expressed as a percentage of the baseline reading for each individual animal. Data are also shown for TPG alone (first column). τ denotes *p*<0.05 versus TPG. * denotes *p*<0.05 for the comparisons shown.

*C. septicum* is a primary contributor to the aetiology of atraumatic gas gangrene [15]. However, its effects on microvascular perfusion have not been examined. Therefore, we used a comparable approach to that used with *C. perfringens*, and applied supernatants of wild-type *C. septicum* (JIR6086) to the cremasteric microvasculature. Similar to the response induced by *C. perfringens*, culture supernatants from *C. septicum* induced a significant microvascular perfusion deficit over a 60 min time course (**Figure 5**). To assess the role of the *C. septicum* α-toxin (a different protein than the similarly named toxin secreted by *C. perfringens*), we compared the perfusion deficit induced by wild-type supernatant with that induced by supernatant from a *csa* mutant (JIR6111) deficient in α-toxin production, and with an isogenic α-toxin-complemented derivative (JIR6146). The relative α-toxin titres for these three strains are shown in **Table 2**. No detectable hemolytic activity was observed for the α-toxin mutant (JIR6111) on horse blood agar. However, as previously observed [17], α-toxin activity was restored to levels above wild-type in the strain complemented for α-toxin (JIR6146), presumably reflecting the multi-copy nature of the complementation plasmid. Exposure of tissues to supernatants from the α-toxin mutant resulted in a mean perfusion at 60 min of 29.4±7.3 mm/mm^2^, versus 17.3±6.5 mm/mm^2^ in response to wild-type *C. septicum* supernatant (*p* = 0.057, when analyzed as % reduction from baseline) (**Figure 6**). In contrast, in tissues exposed to supernatants from the α-toxin-complemented strain (JIR6146), microvascular perfusion was almost entirely absent by 60 min, a substantially greater effect than that seen in response to the wild-type *C. septicum* supernatants (**Figure 6**). This differential response may be explained by the higher α-toxin titre in the complemented strain (**Table 2**).

**Table 2:** Relative *C. septicum* α-toxin (Csa) activity in supernatants from wild-type and mutant *C. septicum* cultures

Strain	Genotype	Ref	Toxin status^a^	*C. septicum* α-toxin (log_2_ (titre))
JIR6086^b^	Wild-type	[17]	α-toxin ^+^	4.0
JIR6111	JIR6086*csaΩermB*	[17]	α-toxin ^−^	<1^c^
JIR6146	JIR6111(pJIR2503)	[17]	α-toxin ^+^	7.0

a *C. septicum* α-toxin

b JIR6086 is a rifampicin resistant derivative of strain BX96; the clinical status of which is not known

c Limit of detectability

## Discussion

The pathology of clostridial myonecrosis is complex and unique. Existing work has demonstrated a role for the cessation of microvascular blood flow in addition to the poorly understood phenomenon of inhibition of leukocyte entry into infected tissues, termed leukostasis [3],[6]. The aim of this study was to use high resolution intravital microscopy to examine the microcirculation directly during exposure to culture supernatants from wild-type and toxin-mutant clostridial strains, and to quantitatively assess microvascular perfusion. The findings indicate that toxins of both *C. perfringens* and *C. septicum* induce rapid and severe reductions in blood flow, observations that correlate with the findings of our previously published examination of the ability of these bacteria to rapidly induce myonecrosis [3]–[5],[17]. Visualization of the microcirculation indicated that blood flow in capillaries was severely reduced. Given the central role of these vessels in the delivery of oxygen and nutrients, this effect would be expected to be an important contributor to the necrosis that occurs in infected tissue. Cell death, as assessed by PI incorporation, was found to occur prior to substantial reduction in tissue perfusion, raising the possibility that this injury was an important contributor to the reduction in perfusion. Platelets and neutrophils were also found to play a role in the response, as were both the α-toxin and perfringolysin O of *C. perfringens*, and the *C. septicum* α-toxin. These findings support our earlier demonstration of the functions of these toxins in inducing clostridial myonecrosis, and provide new insights into the pathogenesis of this syndrome [3]–[5],[17].

The pathogenic mechanisms of the highly virulent *C. septicum* are poorly characterized. Previous studies have demonstrated an essential role for the *C. septicum* α-toxin in myonecrosis [17]. However, the effects of this toxin on the microcirculation have not been examined. Our results provide the first evidence that *C. septicum* toxins have the capability of inducing vascular collapse in a similar manner as toxins from *C. perfringens*. Furthermore, they implicate the *C. septicum* α-toxin as capable of mediating this process, despite the fact that its structure, specificity and biological activity are very different from those of any of the toxins produced by *C. perfringens* type A [20]. However, it was notable that supernatants from α-toxin-deficient *C. septicum* retained the capability of reducing perfusion by approximately 60%, suggesting that other *C. septicum* products such as other hemolysins [21],[22], sialidase [23] or DNAse [24] may have contributed to this response. Additional experiments are needed to determine if there are other *C. septicum* toxin(s) that are involved in mediating the cessation of microvascular blood flow.

In the response to *C. perfringens* infection or exposure to C. perfringens α-toxin, the formation of platelet/leukocyte aggregates and leukostasis have been implicated as being of central importance [3],[7],[12],[25]. In addition, *C. perfringens* α-toxin has been reported to induce interactions between platelets and leukocytes, a process that may underlie leukostasis [26]. In the present study, obvious aggregate formation or thrombosis was not observed in response to superfusion with *C. perfringens* supernatants, although this finding may reflect the different approaches used in these studies. In the model used by Bryant *et al.*, purified clostridial toxins caused reductions in blood flow within less than 5 minutes [7]. Similarly, Alape-Giron *et al*. observed very rapid (within 1–2 min) alterations in the morphology of muscle fibers upon exposure to α-toxin, thrombus formation within 2–5 min, and complete cessation of blood flow within 15–20 mins [12]. In contrast, the response in the present study was much more gradual and associated with minimal changes in muscle morphology. It is likely that these divergent observations are due to differences in the toxin activity present in culture supernatants versus preparations of purified α-toxin. Despite this difference, platelet depletion was partially protective against the reduction in blood flow induced by *C. perfringens* supernatants. These data are consistent with previous findings in which the anti-thrombotic agent heparin was protective against α-toxin-induced vascular collapse [7], and support the hypothesis that platelets are important contributors to the *C. perfringens*-induced deficit in microvascular perfusion.

The role of leukocytes is one of the least well understood aspects of this response. The inability of leukocytes to effectively infiltrate *Clostridium*-infected tissues is a hallmark of the disease pathology [3], but it remains unclear if this is a contributing factor to myonecrosis or an epiphenomenon. Nonetheless, examination of the microvasculature has shown that neutrophil depletion protects against reductions in blood flow induced by *C. perfringens* α-toxin [7]. This finding is supported by the results of the present study, in which neutrophil depletion was protective against the microcirculatory collapse induced by *C. perfringens* supernatants. In studies of ischemia/reperfusion injury, the neutrophil has been shown to contribute to reductions in microvascular perfusion via luminal obstruction of microvessels [27]–[29]. In addition, α-toxin, via platelet GPIIb/IIIa and P-selectin (CD62P), can induce formation of platelet-leukocyte aggregates, which could also act to reduce microvascular perfusion [7],[26]. These processes provide potential mechanisms for the contribution of neutrophils to the defect in microvascular perfusion induced by *C. perfringens*. However, these data must also be viewed in light of recent work examining the effect of neutrophil depletion on *C. perfringens*-induced myonecrosis. O'Brien *et al*. showed that in animals infected with large inoculates of *C. perfringens*, neutrophil depletion failed to protect against myonecrosis [18]. This finding indicates that under conditions where large numbers of live bacteria are present, *C. perfringens* is capable of inducing fatal myonecrosis irrespective of the potential perfusion-maintaining effect afforded by neutrophil depletion.

Many studies implicate α-toxin as being of central importance in *C. perfringens*–associated myonecrosis [2],[4],[12],[30]. In the present study, analysis of the molecular basis of the vascular response to *C. perfringens* confirmed the previously observed capability of the α-toxin to induce vascular collapse [7],[12]. This was shown using α-toxin-deficient *C. perfringens* mutants, which were less effective at reducing perfusion than wild-type *C. perfringens*, and by complementation of an α-toxin and perfringolysin O-deficient strain with α-toxin, which restored the ability to disrupt perfusion to levels not different from that of the wild-type strain. However, these experiments also provided evidence suggesting a role for perfringolysin O in reducing perfusion. Firstly, supernatants from perfringolysin O-deficient strains caused substantially less vascular compromise than those from wild-type strains, although this result may have been complicated by the comcomitant reduction in α-toxin activity in this mutant. Secondly, supernatants from α-toxin/perfringolysin O-double mutant strains complemented with perfringolysin O were capable of reducing microvascular perfusion in some mice, a response not seen in response to supernatants lacking both toxins. These data suggest a novel activity for this toxin, although further work is required to confirm this hypothesis. It has been demonstrated that perfringolysin O contributes to the escape of *C. perfringens* from the macrophage phagosome, is a primary mediator of *C. perfringens*-mediated macrophage cytotoxicity, and can activate TLR4, indicating that perfringolysin O can make important contributions to *C. perfringens*-induced pathology independently of the α-toxin [14],[31]. However, analysis of the virulence of mutant *C. perfringens* strains lacking both α-toxin and perfringolysin O provides clear evidence that the pathology of *C. perfringens* infection is most severe in the presence of both toxins [3],[5],[14].

In cases of clinical *C. perfringens* infection, the toxin levels present in the tissues are unknown. Given this situation, the relative merits of examining the effects of purified toxins versus culture supernatants are difficult to assess. In the present experiments, the rationale for the use of culture supernatants is that it allowed assessment of the tissue response to the complete set of clostridial toxins, an approach that is likely to more accurately model clinical infection. Furthermore, it also allowed the assessment of the effect of removal of single *C. perfringens* gene products from the supernatant. However, it must be noted that the genetic approaches used in this study produced some unexpected effects. In the *C. perfringens* experiments, the α-toxin activity in the perfringolysin O mutant JIR4069 was lower than that in the wild-type strain. Therefore, the possibility cannot be excluded that alterations in perfusion associated with exposure to this strain are due not only to the absence of perfringolysin O, but also to its lower α-toxin activity. Similarly, in the *C. septicum* studies, plasmid-mediated complementation of the *C. septicum* α-toxin mutant resulted in a greater level of toxin activity than that in the wild-type strain. Nevertheless, the level of perfusion defect observed in response to the three *C. septicum* strains examined correlated with the level of α-toxin activity, supporting the concept that the *C. septicum* α-toxin is capable of inducing a reduction in microvascular perfusion.

Vascular leukostasis and the absence of neutrophils is a characteristic of clinical cases of clostridial myonecrosis, but the mechanisms of leukostasis and its contribution to the process of myonecrosis remain unclear. Since the initial trauma associated with *C. perfringens* gangrene cannot be replicated in experimental *C. perfringens* infections, high inoculates are required to induce myonecrosis; at lower infection rates, neutrophil infiltration proceeds unhindered [4],[18]. Furthermore, purified α-toxin has been shown capable of inducing adhesion molecule expression and leukocyte recruitment in a vascular bed in which blood flow was presumably maintained [13]. From these findings it could be argued that if neutrophils are able to enter the infected tissue efficiently before cessation of microvascular perfusion, they are then able to participate in clearing the infection. However, in the present study we observed extensive cell death prior to the most severe reductions in blood flow. It is possible that endothelial cells were irreversibly injured by exposure to the clostridial toxin mixtures. This hypothesis is highly relevant to the process of neutrophil recruitment in that leukocyte transmigration across the endothelium has been shown to require active responses from endothelial cells [32],[33]. It is likely that severely injured endothelial cells would be unable to undergo the cytoskeletal rearrangements necessary to allow transmigration. Therefore, the possibility that leukostasis in part reflects a reduction in efficiency of leukocyte transmigration due to endothelial injury cannot be discounted.

Taken together, these data are consistent with a model of *C. perfringens*-induced myonecrosis in which both α-toxin and perfringolysin O contribute to the reduction in microvascular perfusion. The data also indicate a potential role for injury of local cells, in addition to roles for circulating platelets and neutrophils. Although it was not possible to clarify the influence of *C. perfringens* products on leukostasis, it is clear if a comparably severe reduction in perfusion occurred during clinical infection, then this would prevent delivery of large numbers of leukocytes to the affected site.

## Methods

### Antibodies and Reagents

Antibodies used in these experiments were RB6-8C5 (purified from supernatant) and rabbit anti-thrombocyte serum (Accurate Chemical & Scientific). Sodium fluorescein, PI and all other reagents were purchased from Sigma Chemical Co. (St. Louis, MO), unless otherwise stated.

### Bacterial culture and toxin assays

The genotypes and toxin production levels of clostridial strains are shown in **Tables 1** and **2**. *C. perfringens* culture supernatants were prepared as previously described except that the supernatants were filter-sterilized [4]. *C. perfringens* α-toxin and perfringolysin O were assayed as previously described [34]. *C. septicum* cultures were grown in TPG medium to a turbidity of 1.5 at 600 nm, then centrifuged at 10,016×*g* for 15 min and the supernatants collected, filter sterilized, aliquoted and stored at −70°C. Supernatant samples were then assayed for α-toxin activity as previously described [17].

### Preparation of cremaster muscle for intravital microscopy

Male BALB/c mice were bred in-house (Monash University Animal Services) and housed in conventional conditions. All animal experimentation was approved by the Monash University Animal Ethics ‘B’ Committee. The mouse cremaster muscle was prepared for intravital microscopy as described previously [35]. Briefly, mice were anesthetized with ketamine hydrochloride (150 mg/kg; Pfizer, West Ryde, Australia) and xylazine (10 mg/kg; Troy Laboratories, Smithfield, Australia) by intraperitoneal injection. The left jugular vein was cannulated to administer additional anesthetic and fluorochromes. Catheters were not heparinized to avoid obscuring potential thrombotic effects of clostridial products. Animals were maintained at 37°C on a thermocontrolled heating pad. The cremaster muscle was exteriorized onto an optically-clear viewing pedestal. The muscle was cauterized longitudinally and held flat against the optical window via attachment of silk sutures. The tissue was kept warm and moist by superfusion of warmed bicarbonate buffered saline (pH 7.4), and covered with a coverslip held in place with vacuum grease.

The cremasteric microcirculation was visualized using an intravital microscope (Axioplan 2 Imaging; Carl Zeiss, Australia) with a X 20 objective lens (LD Achroplan 20X/0.40 NA, Carl Zeiss, Australia) and an X 10 eyepiece. Brightfield images of the preparation were visualized using a colour video camera (Sony SSC-DC50AP, Carl Zeiss, Victoria, Australia), and fluorescence images were visualized using a low-light video camera (Dage-MTI IR-1000; SciTech, Preston South, Victoria, Australia). All images were recorded for playback analysis using a videocassette recorder (Panasonic NV-HS950, Klapp Electronics, Victoria, Australia). The number of adherent leukocytes within 100 µm post-capillary venule regions was quantitated as described previously [35]. This intravital microscopy approach was selected to also enable examination of the effects of clostridial toxins on leukocyte-endothelial cell interactions. However, it was found that treatment of muscle with diluted TPG alone elevated leukocyte interactions above that in buffer-treated mice. In addition, leukocyte adhesion in muscles exposed to JIR325 supernatant was not significantly increased above that in mice treated with TPG alone (data not shown). Therefore leukocyte rolling and adhesion were not assessed in further groups.

### Analysis of functional capillary density (FCD)

After a 20 min stabilization period following cremaster exteriorization, an initial assessment of the microcirculation was performed in the exteriorized cremaster muscle. Intravenously administered sodium fluorescein, imaged via epifluorescence (excitation wavelength–460 nm, emission wavelength–515 nm) was used to delineate perfused microvessels. A single bolus of sodium fluorescein (5 µL of 10 mg/ml in saline) was injected intravenously, and fluorescence images of five adjacent areas of tissue rapidly recorded on video for subsequent analysis. This low molecular weight fluorescent marker was selected as it was rapidly cleared from the circulation thus allowing assessment of the functional state of the microcirculation at multiple time points in the same animal. The cremaster was then superfused for 60 min with supernatant from various strains of *C. perfringens* or *C. septicum*, diluted 1∶1 in bicarbonate superfusion buffer, and perfusion re-assessed at the end of the experiment in identical fashion. In some experiments, additional readings were performed after 30 min of supernatant superfusion.

To quantitate functional capillary density, single video frames were captured using *Adobe Premiere* software, and functional capillary density determined using *Adobe Photoshop*, as previously described [36]. Briefly, the captured video frames were opened, converted to RGB mode, and a new layer generated. The *Pencil* tool was then used to trace a line (5 pixel width) over the capillaries containing fluorescent material. The *Magic Wand* tool was used to select the traced lines, and the *Histogram* command used to quantitate the number of pixels in the traced line. Based on the width of the traced line and the resolution and magnification of the image, the length of the perfused capillaries was calculated and expressed per unit area (mm/mm^2^) [36].

To assess the role of neutrophils in the microvascular perfusion response, mice were pre-treated with anti-Gr-1 antibody (RB6-8C5, 150 µg/mouse i.p.) 4 hrs prior to the experiment [19]. Similarly, to examine the role of platelets, platelet depletion was achieved via injection of rabbit anti-mouse thrombocyte serum (15 µL, i.p.) 4 hrs before exposure to *C. perfringens* supernatants [19].

### In vivo analysis of cellular injury

Cell death in the exteriorized muscle preparation was assessed using PI staining, as previously described [37]. PI labels the nuclei of cells with disrupted cell membranes, but not healthy cells. The muscle was superfused with PI (1.0 µM, in superfusion buffer) and PI-stained nuclei visualized via epifluorescence (excitation wavelength–535 nm, emission wavelength–617 nm). Images of two randomly-selected regions within the muscle were captured at defined intervals throughout the experimental period. The number of PI-positive nuclei per frame was quantified and expressed as (cells/field).

### Statistical Analysis

All data are displayed as mean±SEM. Student's *t* tests or one way ANOVA was performed to compare experimental groups. *P* values <0.05 were considered significant.

## Supporting Information

Video S1 Video showing normal microvascular perfusion in the cremaster muscle 60 mins after commencement of TPG superfusion. Several areas of muscle are shown shortly after intravenous administration of sodium fluorescein, which labels flowing blood vessels. Functional, perfused capillaries can be detected via the presence of fluorescent (white) material moving through multiple small caliber vessels. The degree of microvascular perfusion in this tissue is consistent with complete perfusion of all capillaries in the muscle.
(7.00 MB MOV)

Video S2 Video showing effect of superfusion with supernatants from wild-type *C. perfringens* (JIR325). Several areas of muscle are shown shortly after intravenous administration of sodium fluorescein. Sixty min after commencing superfusion, the absence of microvascular perfusion is manifest as large areas of muscle containing very few fluorescein-filled microvessels. Sodium fluorescein-associated fluorescence is only apparent in occasional larger arterioles and venules, whereas only few capillaries are perfused. The reduction in the number of perfused capillaries in this experiment is readily apparent by comparison with that in muscles superfused with TPG (Video S1).
(6.47 MB MOV)

Video S3 Video showing effect of superfusion with supernatants from an α-toxin/perfringolysin O mutant (JIR4444) of *C. perfringens*. Several areas of muscle are shown shortly after intravenous administration of sodium fluorescein. After 60 min of superfusion, substantial capillary perfusion is apparent in these tissues (demonstrated by the presence of intravenous fluorescein). Indeed, the degree of capillary perfusion in muscles exposed to supernatant deficient in α-toxin and perfringolysin O is comparable to that in tissues exposed to TPG (Video S1), indicating the combined actions of these toxins in promoting loss of perfusion.
(5.16 MB MOV)
